# Multi-Omics Analysis for Transcriptional Regulation of Immune-Related Targets Using Epigenetic Data: A New Research Direction

**DOI:** 10.3389/fimmu.2021.741634

**Published:** 2022-01-03

**Authors:** Chenshen Huang, Na Zhang, Hao Xiong, Ning Wang, Zhizhong Chen, Zhizhan Ni, Xiaohong Liu, Boxu Lin, Bujun Ge, Bing Du, Qi Huang

**Affiliations:** ^1^ Department of General Surgery, Tongji Hospital, School of Medicine, Tongji University, Shanghai, China; ^2^ Shanghai Key Laboratory of Regulatory Biology, Institute of Biomedical Sciences and School of Life Sciences, East China Normal University, Shanghai, China; ^3^ Shengli Clinical Medical College of Fujian Medical University, Fujian Provincial Hospital, Fujian, China

**Keywords:** chromatin accessibility, ATAC-seq, ChIP-seq, multi-omics analysis, transcription factor

## Abstract

**Background:**

Currently, a comprehensive method for exploration of transcriptional regulation has not been well established. We explored a novel pipeline to analyze transcriptional regulation using co-analysis of RNA sequencing (RNA-seq), assay for transposase-accessible chromatin using sequencing (ATAC-seq), and chromatin immunoprecipitation with high-throughput sequencing (ChIP-seq).

**Methods:**

The G protein-coupled receptors (GPCRs) possibly associated with macrophages were further filtered using a reduced-Cox regression model. ATAC-seq profiles were used to map the chromatin accessibility of the GPRC5B promoter region. Pearson analysis was performed to identify the transcription factor (TF) whose expression was correlated with open chromatin regions of GPRC5B promoter. ChIP-seq profiles were obtained to confirm the physical binding of GATA4 and its predicted binding regions. For verification, quantitative polymerase chain reaction (qPCR) and multidimensional database validations were performed.

**Results:**

The reduced-Cox regression model revealed the prognostic value of GPRC5B. A novel pipeline for TF exploration was proposed. With our novel pipeline, we first identified chr16:19884686-19885185 as a reproducible open chromatin region in the GPRC5B promoter. Thereafter, we confirmed the correlation between GATA4 expression and the accessibility of this region, confirmed its physical binding, and proved *in vitro* how its overexpression could regulate GPRC5B. GPRC5B was significantly downregulated in colon adenocarcinoma (COAD) as seen in 28 patient samples. The correlation between GPRC5B and macrophages in COAD was validated using multiple databases.

**Conclusion:**

GPRC5B, correlated with macrophages, was a key GPCR affecting COAD prognosis. Further, with our novel pipeline, TF GATA4 was identified as a direct upstream of GPRC5B. This study proposed a novel pipeline for TF exploration and provided a theoretical basis for COAD therapy.

## Highlights

- This study provides a novel pipeline to explore transcription factors based on multi-omics data, which is described adequately enough to be repeated and taken further.
- With our pipline, GATA4 was identified to be the direct upstream transcription factor regulating GPRC5B.
- GPRC5B may affect COAD patient prognosis, possibly by interacting with macrophages

## Introduction

Colon cancer, a type of malignant tumor, is the third leading cause of cancer deaths worldwide (1–3). Colon adenocarcinoma (COAD) is the most common pathological subtype of colon cancer. Currently, the prognosis of advanced COAD patients remains poor. Accordingly, a better understanding of the molecular mechanisms involved in COAD is warranted.

In recent years, increasing evidence has revealed that immune infiltration might be an essential factor in COAD patient prognosis (4). Tumor-infiltrating macrophages of mixed origin are an important component of immune infiltration (5). Currently, several studies have indicated that tumor-infiltrating macrophages could impact COAD progression (6, 7). However, an improved understanding of the underlying mechanisms of action is still needed, and a search for potential macrophage-targeted therapeutic options is also required.

G protein-coupled receptors (GPCRs), a group of cell surface signaling proteins, represent the most prominent superfamily of pharmacological targets (8, 9). It has been confirmed that various GPCRs are involved in the progression of tumors (10), including colon cancer (11). Lysophosphatidic acid receptors (12), protease-activated receptor 1 (13), prostaglandin E2 receptors (14), and endothelin receptors (15) have all been identified as key players in colon cancer. Nevertheless, the functions of many GPCRs still remain unclear. Thus, an improved understanding of the involvement of GPCRs in colon cancer formation and progression might contribute to the development of a novel generation of antitumor therapeutics. Additionally, to date, a large number of GPCRs have been reported to influence tumor development through macrophages (16, 17). For example, our previous study demonstrated that LGR4 could maintain protumoral macrophages and thus play a vital role in tumor progression (18). Considering the information presented above, we inferred that macrophage-associated GPCRs could be a potential target for COAD therapy, and we employed bioinformatic methods for a more comprehensive investigation.

Currently, to explore the regulation of a target gene, various algorithms would be applied to transcriptome data for pathway quantization, followed by correlation analysis. If the target gene was statistically correlated with a quantized pathway, its involvement in the regulation of this pathway would be proposed. Besides analysis of transcriptome data from RNA sequencing (RNA-seq) profiles, our study further acquired epigenetic data to explore the target gene’s regulation. Assay for transposase-accessible chromatin using sequencing (ATAC-seq) used Tn5 transposase to determine the nucleosome position and map the open chromatin regions (19, 20). The accessible chromatin sites in the promoter regions reflect the potential binding of transcription factors (TFs). Genes with chromatin accessibility in the promoters were more likely regulated by TFs. ATAC-seq profiles could detect the open chromatin regions of target genes and indicate their regulatory mechanism. Through co-analysis of ATAC-seq and transcriptome data, we could identify potential TFs, whose expressions were significantly correlated with the open promoter regions of the target gene. Next, chromatin immunoprecipitation (ChIP)-seq profiles were obtained to confirm the physical binding of potential TFs and the predicted binding regions.

Here, multi-omics bioinformatics was employed to discover the macrophage-correlated GPCRs that might play a key role in COAD. With transcriptome data from RNA-seq profiles, we explored the GPCRs that might be associated with tumor infiltrating macrophages. GPRC5B was eventually selected based on its clinical value. Moreover, epigenetic data from ATAC-seq profiles were also obtained to explore regulation mechanisms. The above-mentioned results were verified through quantitative polymerase chain reaction **(**qPCR), chromatin immunoprecipitation with high-throughput sequencing (ChIP-seq), and multidimensional databases.

## Materials and Methods

### Ethics

Our study was approved by Tongji Hospital, Shanghai, China (reference number 2018-LCYJ-005). Written consent was obtained from all participants/patients before the study.

### Data Collection

The ATAC-seq profiles of 41 COAD samples were obtained from the NCI Genomic Data Commons (https://gdc.cancer.gov/about-data/publications/ATACseq-AWG). We acquired the Fragments Per Kilobase per Million mapped reads (FPKM) and htseq-count profiles of 514 samples from TCGA database (https://tcga-data.nci.nih.gov/), including 473 COAD samples and 41 solid normal tissue samples. Clinical demographic information of 426 COAD patients was also retrieved. The baseline information of all COAD patients is provided in **Supplemental Table S1**.

### Infiltrating Immune Cells

The normalized gene expression matrix was obtained from the FPKM profiles. Further, based on the signature markers provided by Charoentong et al. (21), the Single Sample Gene Set Enrichment Analysis (ssGSEA) (22, 23) was applied to estimate the tumor-infiltrating immune cells in COAD. The signature markers are all listed in **Supplemental Table S2**. We applied the Wilcoxon rank-test to compare the difference in the abundances of immune cells between COAD samples and normal solid tissue samples.

### Integrative Analysis of GPCRs and Tumor-Infiltrating Macrophage

The list of GPCRs was downloaded from the GPCR NaVa database (http://nava.liacs.nl) (24) and gene expression of recorded GPCRs were retrieved (**Supplemental Table S3**). To explore the GPCRs potentially correlated with tumor-infiltrating macrophages, we performed a Spearman correlation analysis. The filtered GPCRs were further included in the Lasso regression model and the reduced-Cox regression model. Eventually, based on the reduced-Cox model, a nomogram was constructed to predict COAD patient prognosis. Calibration curves were displayed to validate the accuracy and discrimination of the nomogram.

### A Novel Pipeline for TF Exploration

Publicly available datasets were analyzed in this study, including 41 paired ATAC-seq and RNA-seq profiles of the same COAD patients. The sources of these profiles have been described in data collection section. The upstream analysis of ATAC-seq data was completed following the pipeline proposed by M. Ryan Corces et al. And a total of 122872 reproducible peaks were observed in 41 ATAC-seq profiles of COAD patients. In this study, we directly downloaded these peaks from the supplemental data file “cancer type-specific count matrices in normalized counts” (https://gdc.cancer.gov/about-data/publications/ATACseq-AWG).

**Figure 1:**
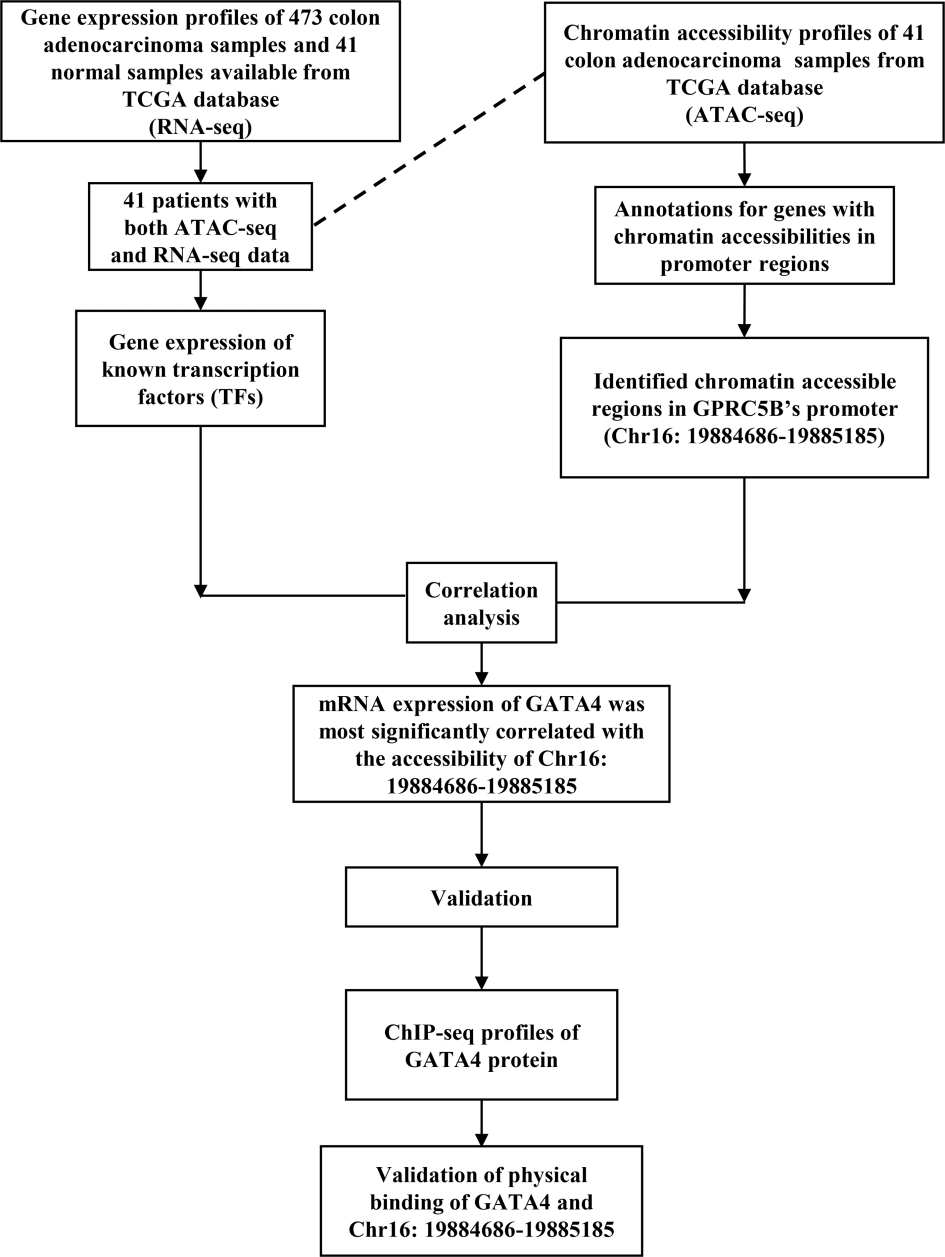
An overview of the novel pipeline of our study.

With the data above, we proposed a novel pipeline to explore TF regulation based on multi-omics data. Our pipeline included: 1) Peak annotation. We used the R package “ChIPseeker” to annotate peaks (*annotatePeak* function with tssRegion from -2000 to 2000, TxDb equal to “TxDb.Hsapiens.UCSC.hg38.knownGene”, and annoDb equal to “org.Hs.eg.db”). We obtained the 45377 peaks which were annotated as “Promoter (<=1kb)” or “Promoter (1-2kb)”. 2) Getting the peaks located in the promoter region of target gene. We first used the R package “GenomicFeatures” to check the gene location of the target gene GPRC5B (*genes* function with x equal to “TxDb.Hsapiens.UCSC.hg38.knownGene”). Then, we search the above 45377 peaks, and we found the target peak (chr16:19884686-19885185) which had an overlap with the gene location of GPRC5B (chr16: 19856691-19886167). 3) Getting the mRNA expression of TFs. We downloaded the list of TFs from the Cistrome database (http://cistrome.org/). Based on the list, we can get the TF mRNA expression from RNA-seq profiles. 4) We used the R package “stats” (*cor.test* function) to perform the Pearson correlation analysis between the TF mRNA expression and the ATAC-seq peak accessibility of the target peak (chr16:19884686-19885185). The correlation threshold was set as an absolute value of r > 0.2, p < 0.01. In this study, the TF GATA4 expression was found to be most highly correlated with the target peak (chr16:19884686-19885185). 5) We used the Cistrome to browse the GATA4 ChIP-seq data (*Cistrome Data Browser* function with species equal to “Homo sapiens”, and factors equal to “GATA4”), and we can check the overlap between the target peak (chr16:19884686-19885185) and the peaks of GATA4 ChIP-seq data. **Figure 1** presents an overview of the pipeline of our study.

### Co-Analysis of ATAC-Seq and RNA-Seq Profiles

Chromatin accessibility analysis was performed based on ATAC-seq profiles. Peak regions over chromosomes were visualized through the R package karyoploteR (25). We also applied ChIPseeker (26) to map the tagMatrix, indicating the locations of peaks around the transcription start site (TSS) regions. The peaks near the TSS regions were annotated by TxDb. Hsapiens. UCSC. Hg38. knownGene. For visualization, we used a pie plot to better show the relationship between peak locations and promoter regions.

To explore the TF directly upstream of GPRC5B, the Pearson correlation analysis was performed for TF mRNA expression and chromatin accessibility of the GPRC5B promoter region. The potential TFs were further filtered by ChIP-seq profiles of colon cancer cells, which were obtained from the Cistrome database (http://cistrome.org/) (27, 28).

### Multi-Database Validation

To minimize the bias of using a single database, we validated our results using multiple databases, including: Gene Expression Omnibus (GEO, ID: GSE85001, https://www.ncbi.nlm.nih.gov/geo/), Timer 2.0 database (http://timer.comp-genomics.org/) (29, 30), GEPIA database (http://gepia.cancer-pku.cn/) (31), Human Protein Atlas database (https://www.proteinatlas.org/) (32, 33), and LinkedOmics database (https://linkedomics.org/) (34). We also used four different algorithms (EPIC (35), XCELL (36), TIMER (37), and MCP-counter (38) algorithms) to confirm the significant correlation between tumor-infiltrating macrophages and GPRC5B. The EPIC and TIMER algorithms are partial deconvolution algorithms, while the XCELL and MCP-counter algorithms are scoring methods based on a set of marker genes (39).

### qPCR Validation

We additionally validated the mRNA expression level of GPRC5B using fresh frozen tissue samples. COAD samples and paired normal tissues were obtained from 28 patients from Tongji Hospital, Shanghai, China. TRIzol reagent (Magen, R4801-01) was used for RNA extraction following the manufacturer’s instructions. After reverse transcription, qPCR was performed using cDNA, SyberGreen (Yeasen, 11200ES08) and human GPRC5B primers (Forward: ACAATGCAGCTCTCCGAACAG, Reverse: TGATACACGTTGCTTCTAAACGG). The amplification program was set as follows: 95°C for 210 sec; 40 cycles at 95°C for 210 sec, 58°C for 30 sec, 63°C for 20 sec; melting curve from 58°C to 95°C. Every sample in the qPCR experiment was repeated in triplicate. Additionally, human GAPDH was selected as the internal control (Forward: GGAGCCAAAAGGGTCATCATCTC, Reverse: TGATGGCATGGACTGTGGTCATG). A paired *t*-test was applied to screen the significant differences.

### Statistical Analysis

Statistical significance was set at p < 0.05. The correlation threshold was set as an absolute value of r > 0.2, p < 0.01 in Pearson or Spearman analysis. Variable normality was checked using Shapiro-Wilk normality test. For nonnormally distributed variables, the Wilcoxon rank-test was used for two independent group comparisons, while the Student’s t-test was used to compare normally distributed variables. Paired data which were normally distributed were analyzed by paired t-test. In Kaplan-Meier survival analysis, to prevent the bias caused by non-tumor-related death, we obtained results only from patients who had a follow-up time of more than 90 days. The optimal cutoffs for Kaplan-Meier survival analysis were determined by R package survminer (Version 0.4.9; https://CRAN.R-project.org/package=survminer). The differentially expressed genes were identified by R package edgeR (Version 3.28.1), and the statistical significance was set at adjusted p < 0.05. R (version 3.5.1; www.r-project.org) was the main analysis software.

## Results

### Quantitation of Macrophages in COAD

**Figure 2:**
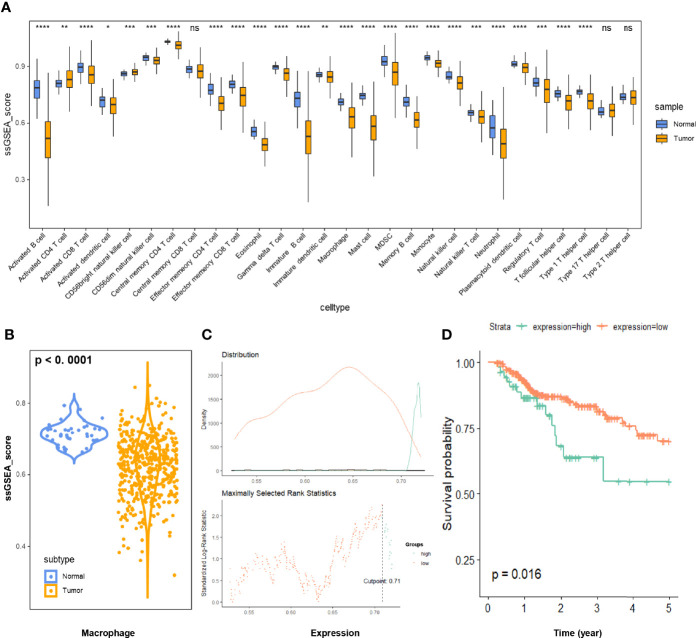
**(A)** Immune infiltration of 28 immune cell subtypes was quantitated *via* the ssGSEA method in 473 COAD samples and 41 normal tissue samples. **(B)** The relative macrophage infiltration was significantly downregulated in COAD tissues (p < 0.0001). **(C)** Illustration of the optimal cutoff point identification for survival analysis in **(D)**. The cutoff point with the maximum standardized Log-rank statistical value was regarded as the optimal cutoff point. **(D)** Kaplan-Meier plot of 5-year survival of COAD patients with high vs. low ssGSEA scores of macrophage infiltration. The COAD patients with higher macrophage infiltration displayed poorer prognosis (p < 0.05). COAD, colon adenocarcinoma; ssGSEA, single sample gene set enrichment analysis. * p ≤ 0.05; ** p < 0.01; *** p < 0.001; **** p < 0.0001; ns, not significant.

We estimated the scaled proportion of tumor-infiltrating immune cells using the ssGSEA method to render the immune cells of each COAD patient comparable (**Figure 2A**). To make the results more reproducible and reliable, the whole ssGSEA process followed the signature markers from Charoentong et al. The detailed macrophage subsets, such as M0, M1, and M2, would be further analyzed in the final multi-database validation part. According to the violin plot in **Figure 2B**, total macrophage expression was significantly decreased in COAD samples. Kaplan-Meier plot of 5-year survival indicated that the COAD patients with higher macrophage infiltration displayed poorer prognosis (p < 0.05, **Figures 2C, D**).

### Identifying GPCRs Significantly Correlated With Macrophages in COAD

To explore the potentially relevant GPCRs, we first filtered the recorded GPCRs by Spearman correlation analysis. The list of known GPCRs was obtained from the GPCR NaVa database, and GPCR expressions were retrieved from the RNA-seq profiles. Eventually, correlation analysis between GPCRs and macrophage expression was performed, identifying 190 GPCRs for further analysis (**Supplemental Table S4**).

### Identification of the Potential Prognostic Biomarker GPRC5B

**Figure 3:**
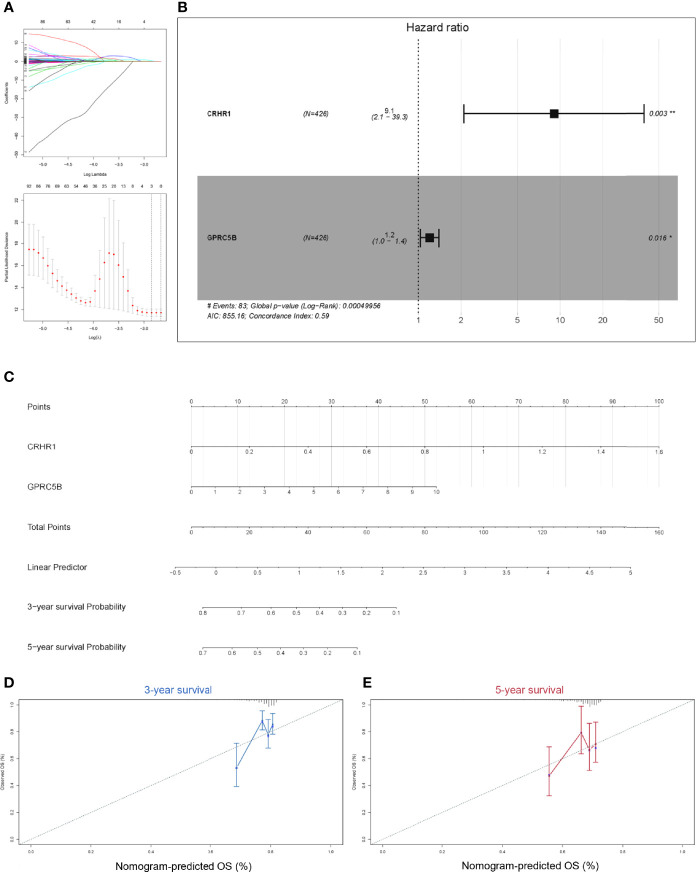
**(A)** To prevent the bias caused by overfitting, LASSO regression was applied. **(B)** Based on the LASSO regression results, the reduced multi-Cox regression model was constructed. CRHR1 (p = 0.003) and GPRC5B (p = 0.016) were shown to be potentially correlated with COAD prognosis. **(C)** A nomogram based on the reduced-Cox model in **(B)** was constructed (p = 0.0005, AIC = 855.16, C-index = 0.6). **(D, E)** Calibration curves of 3-year survival **(D)** and 5-year survival **(E)** validated the acceptable accuracy of the model. LASSO, least absolute shrinkage and selection operator; CRHR1, corticotropin releasing hormone receptor 1; GPRC5B, G protein-coupled receptor class C group 5 member B; COAD, colon adenocarcinoma.

Considering that macrophages might influence COAD patient prognosis in a GPCR-related way, we aimed to identify a group of GPCRs correlated with macrophages and presented as prognostic predictors. Thus, all GPCRs obtained in the aforementioned results were further processed using the Cox regression model. We utilized LASSO regression to prevent overfitting (**Figure 3A**). According to the LASSO regression results, CRHR1 and GPRC5B were regarded as eligible and were included into the final reduced-Cox regression model (C-index = 0.59, p < 0.05, **Figure 3B**). The reduced-Cox regression model indicated that CRHR1 (p = 0.003) and GPRC5B (p = 0.016) might evaluate the prognosis of COAD effectively. Furthermore, a nomogram was also constructed (**Figure 3C**), and the calibration curves indicated acceptable accuracy (**Figures 3D, E** **)**.

**Figure 4:**
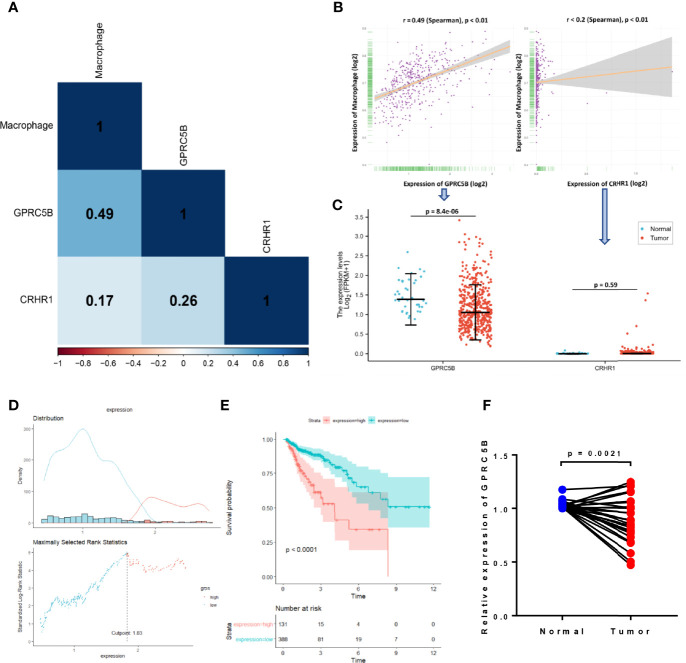
**(A)** Correlation heatmap of macrophages and CRHR1 and GPRC5B expression. **(B)** GPRC5B was significantly correlated with macrophages (r = 0.49, Spearman, p < 0.01). CRHR1 was statistically, but weakly, correlated with macrophages (r < 0.2, Spearman, p < 0.01). **(C)** GPRC5B was a differentially expressed gene in COAD samples (p = 8.4e-06), whereas CRHR1 was only detected in a small number of samples. **(D)** An illustration of the optimal cutoff point identification for survival analysis in **(E)**. **(E)** GPRC5B was a potential prognostic risk factor in COAD patients (p < 0.0001). **(F)** For qPCR validation, clinical samples of 28 COAD tissues and paired normal tissues were acquired from Tongji Hospital, Shanghai, China. GPRC5B was significantly downregulated in COAD tissues (p = 0.0021). CRHR1, corticotropin releasing hormone receptor 1; GPRC5B, G protein-coupled receptor class C group 5 member B; COAD, colon adenocarcinoma.

Although both CRHR1 and GPRC5B might be important prognostic factors in COAD, we found that GPRC5B was more highly correlated with tumor-infiltrating macrophages [r = 0.49 (Spearman), p < 0.05, **Figures 4A, B**]. Further, as shown in **Figure 4C**, CRHR1 was only detected in part of the RNA-seq profiles, while GPRC5B was widely expressed in COAD patients. Collectively, we identified GPRC5B as a potential macrophage-related biomarker in COAD patients. More specifically, GPRC5B was a prognostic risk factor in COAD (**Figures 4D, E** **)**.

### Verification of GPRC5B Expression in COAD *via* qPCR

First, we applied the R package EdgeR (40) to the htseq-count profiles, identifying GPRC5B as a differentially expressed gene in COAD (p < 0.05, **Figure 4C**). To minimize the bias caused by bioinformatics, we also obtained clinical samples from Shanghai Tongji Hospital and performed qPCR on COAD tissues and the paired normal tissues from 28 patients. The qPCR result was consistent with our bioinformatic findings, indicating that GPRC5B was significantly downregulated in COAD tissues (**Figure 4F**).

### Novel Pipeline Identified GATA4 as a Direct TF for GPRC5B

**Figure 5:**
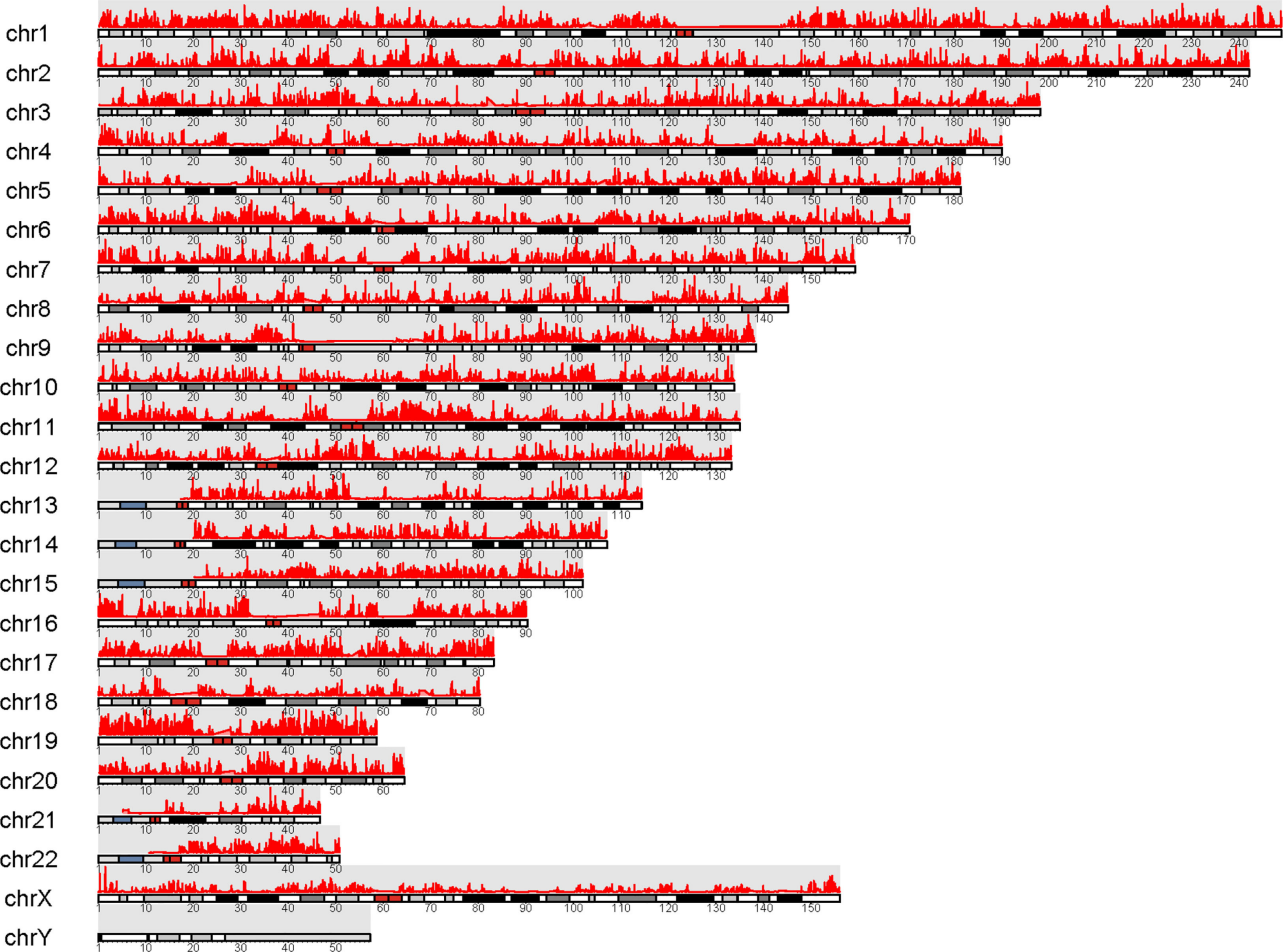
Visualization of peaks (marked in red) over chromosomes. Chromatin accessibilities were widely present over the whole genome.

**Figure 6:**
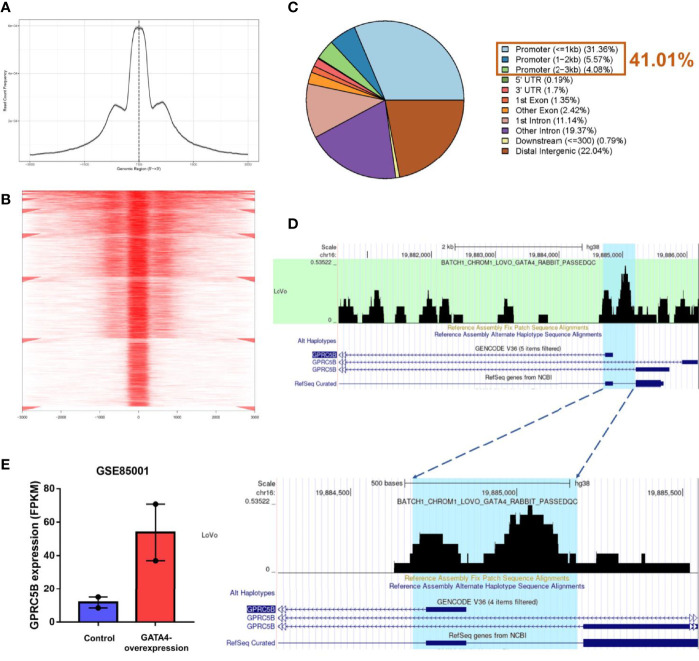
**(A)** Visualization of read count frequency of peaks revealed that they were primarily located around the TSS. **(B)** Peaks are mapped to the TSS regions and further aligned in a tagMatrix, indicating that the majority of chromatin accessibilities were near the TSS. **(C)** Pie plot reveals that most of the open chromatin regions were located in promoters. **(D)** GATA4 ChIP-seq data show the strong overlap between the GATA4 protein binding regions and the predicted regions (highlighted in blue). GATA4 could physically bind to the GPRC5B promoter at chr16: 19884686-19885185. **(E)** According to the GEO database (GSE85001), GPRC5B was significantly upregulated in the GATA4-overexpression group. TSS, transcription start site; ChIP-seq, chromatin immunoprecipitation sequencing; GPRC5B, G protein-coupled receptor class C group 5 member B; GEO, Gene Expression Omnibus.

To further explore the regulation of GPRC5B, we combined ATAC-seq and RNA-seq profiles for co-analysis. It is well-known that TFs regulate genes by binding to the open regions around the promoter. Thus, we acquired the chromatin accessibility landscape of COAD patients from 41 ATAC-seq profiles. **Figure 5** showed that accessibilities were widely presented across the genome. Most open chromatin regions located around TSS regions (**Figures 6A, B** **)**. For visualization, the pie plot indicated that the open chromatin regions were primarily located in the promoter regions (41%, **Figure 6C**). Across samples, we identified chr16:19884686-19885185 as the reproducible open chromatin region in the GPRC5B promoter.

Thereafter, according to the TF list from CISTROME database, we retrieved a TF expression matrix from the FPKM profiles. Correlation analysis was applied to TF expression and the identified accessible promoter regions (chr16:19884686-19885185) (**Supplemental Table S5**). GATA4 expression was found to be most highly correlated with the GPRC5B open promoter regions (p < 0.01), indicating that the TF GATA4 might regulate GPRC5B.

For validation, we then acquired the GATA4 ChIP-seq data from CISTROME. **Figure 6D** shows that the GATA4 protein specifically bonded to the identified promoter regions (Blue area, chr16:19884686-19885185) in colon cancer cells.

After confirming the physical combination between GATA4 and the GPRC5B promoter, we downloaded the RNA-seq profiles of GATA4-overexpressed cells from GEO (GSE85001). GPRC5B was identified as a differentially expressed gene and found to be upregulated in the treatment group (**Figure 6E**).

**Figure 7:**
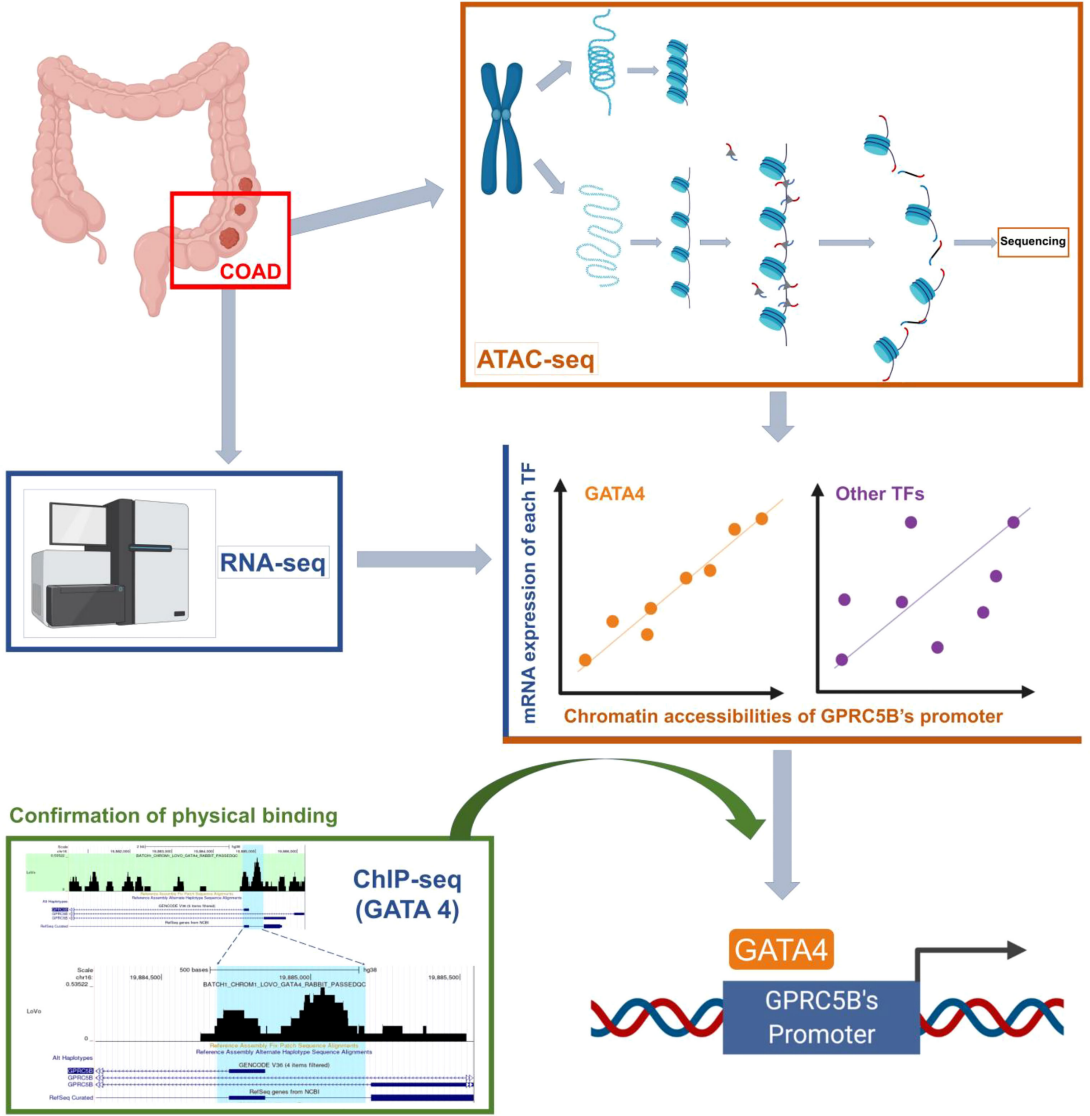
Visualization of the detailed steps of our novel pipeline.

Collectively, the results showed that the TF GATA4 could bind to the GPRC5B promoter regions, regulating GPRC5B expression. Detailed steps of the novel pipeline above are visualized in **Figure 7**.

### Multiple Database Validation

**Figure 8:**
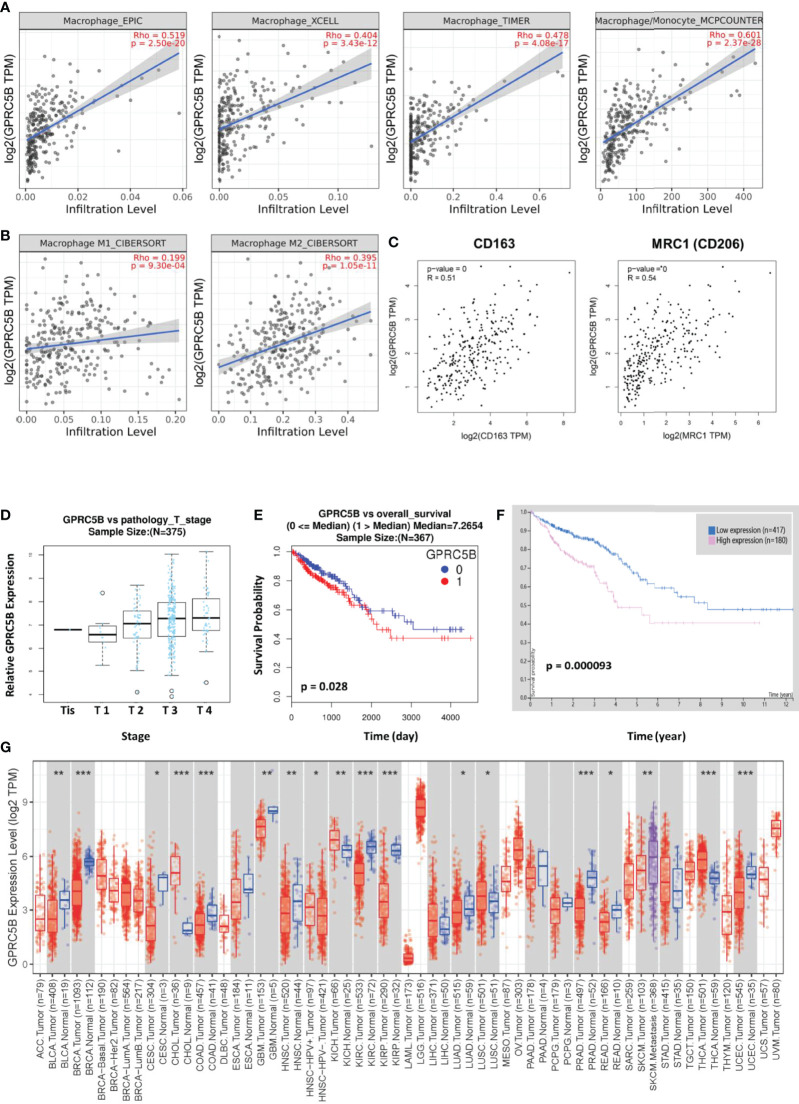
**(A)** Based on the EPIC, XCELL, TIMER, and MCPcounter algorithms, GPRC5B was closely correlated with tumor-infiltrating macrophages in COAD. **(B)** The CIBERSORT algorithm indicated a closer correlation between GPRC5B and M2 macrophages (r = 0.395, p < 0.01), compared with M1 macrophages (r = 0.199, p < 0.01). **(C)** The GEPIA database showed that GPRC5B was significantly correlated with the M2 macrophage surface marker CD163 (r = 0.51, p < 0.01) and MRC1 (r = 0.54, p < 0.01). **(D)** The LinkedOmics database confirmed that GPRC5B was gradually upregulated following the progress of COAD development through different tumor stages. **(E)** The LinkedOmics database indicated that GPRC5B could be a potential risk factor for COAD (p < 0.05). **(F)** The Human Protein Atlas database indicated that GPRC5B could be a potential risk factor for COAD (p < 0.05). **(G)** The Timer 2.0 database showed the mRNA expression level of GPRC5B across various tumor types. GPRC5B was significantly downregulated in COAD samples (p < 0.001). GPRC5B, G protein-coupled receptor class C group 5 member B; COAD, colon adenocarcinoma; TPM, Transcripts per million. * p ≤ 0.05; ** p < 0.01; *** p < 0.001.

To prevent bias due to the use of a single database, multiple databases were used for validation. We confirmed that GPRC5B was significantly correlated with tumor-infiltrating macrophages using four algorithms (EPIC, XCELL, TIMER, and MCP-counter, **Figure 8A**). To be more exact, based on the CIBERSORT algorithm, we found that GPRC5B was more highly correlated with M2 macrophages (r = 0.395, p < 0.01, **Figure 8B**). Similar results were obtained from the GEPIA database (**Figure 8C**), indicating that GPRC5B was correlated with the surface marker CD163 (r = 0.51, p < 0.01) and MRC1 (r = 0.54, p < 0.01) of M2 macrophages.

Furthermore, according to the LinkedOmics database, there was a significant increase in GPRC5B expression with advancing T stage (**Figure 8D**). Patients with high GPRC5B expression tended to have poorer prognosis (**Figure 8E**). The Human Protein ATLAS database also confirmed the prognostic value of GPRC5B (**Figure 8F**). Moreover, according to Timer 2.0 database, GPRC5B was identified as a differentially expressed gene in various tumor types, including COAD (**Figure 8G**).

## Discussion

COAD is one of the most fatal malignant tumor types worldwide. In recent years, an increasing number of studies have indicated that tumor-infiltrating immune cells might play an important role in cancer development and progression (41–43). However, further study of the underlying mechanisms is still warranted. Conversely, although various GPCRs have been proven to be involved in tumor progression, the functions of many GPCRs remain unclear. As cell surface proteins, GPCRs could regulate a wide range of physiological processes and have always been important targets for drug development. Thus, we discovered that macrophage-associated GPCRs showed prognostic value in COAD. Our study aimed to explore potential pharmacological targets for COAD.

In this study, we found that GPRC5B was a key GPCR affecting COAD patient prognosis and could be a novel target of antitumor therapeutics. Also, considering the potential value of GPRC5B, we decided to further explore the regulation of GPRC5B through integrated bioinformatics. Combining RNA-seq and ATAC-seq profiles together, we identified GATA4 as a direct upstream TF of GPRC5B. The results above were verified through qPCR, ChIP, cell experiments, and multidimensional database validations.

GPRC5B belongs to type 3 GPCR family, characterized by a signature seven-transmembrane-domain motif. First identified in 2000 by Hans Brauner-Osborne and Povl Krogsgaard-Larsen (44), GPRC5B is currently an orphan heterotrimeric GPCR. It has been reported to modulate insulin secretion, and it might be associated with type 2 diabetes (45). Additionally, Carvalho et al. revealed that GPRC5B might regulate the membrane availability of the prostacyclin receptor (46). Furthermore, some studies indicated that GPRC5B might be involved in the regulation of obesity-associated inflammatory response and macrophage infiltration (47, 48). Some studies have indicated the role of GPRC5B in cancer, while its specific molecular mechanisms remain largely unknown (49–51). Our findings have supported the clinical value of GPRC5B in patients with COAD.

We identified GPRC5B as a differentially expressed gene in COAD through RNA-seq and qPCR of clinical samples. GPRC5B was significantly downregulated in COAD patients, while its expression would increase with the increase in tumor stages. Considering that GPRC5B was known as a cell surface protein, its expression pattern would make it an ideal pharmacological target. Next, according to the integrated analysis of GPRC5B and tumor-infiltrating immune cells, we also showed that GPRC5B was significantly associated with macrophages. As this correlation was primarily based on statistical methods, and the macrophages were quantized through the ssGSEA algorithm, we acquired multiple algorithms for validation. The classical algorithms, including EPIC, XCELL, TIMER, MCP-counter, and CIBERSORT, were all employed in this study to validate the result of ssGSEA algorithm. With the knowledge that macrophages are important in COAD development and progression, we hypothesized that the interactions between GPRC5B and tumor-infiltrating macrophages, potentially type M2, might be important in COAD and further affect the prognosis of patients with COAD. Based on the results above, we found that GPRC5B is a potential therapeutic target for COAD.

Moreover, a novel pipeline of multi-omics analysis was proposed in this study. Here, we applied this novel pipeline to explore the regulation of GPRC5B, identifying GATA4 as its direct upstream TF. The detailed workflow is displayed in **Figure 1**. In recent years, there have been various studies, which followed the classic pipeline to solve similar questions. However, most of these studies could only prove the correlation between the target genes and the algorithm-quantized pathways, primarily based on transcription data and Pearson/Spearman correlation analysis. Although multi-omics databases were used for validation, it was still hard to further explore the underlying mechanism. For example, Pearson/Spearman analysis could only tell the correlation degree based on statistics, instead of biological significance. Also, there were currently plenty of quantization algorithms, transferring the mRNA expression matrix into immune cell fractions, microenvironment scores, pathway expressions, and other key parameters. There was no doubt that these algorithms were effective and convincing. However, if we primarily focus on mRNA level and statistical screening, some important biological process might be ignored. To solve these problems, many researchers would apply laboratory experiments to confirm biological significances.

Considering the reasons above, we combined ATAC-seq, RNA-seq, and ChIP-seq profiles together for integrated analysis. ATAC-seq used Tn5 transposase to map the open chromatin regions, indicating the potential binding sites for TFs. It was known that genes with chromatin accessibility in the promoter regions are more likely to be regulated by TFs. The ChIP-seq profiles could ensure the physical binding between a specific TF and the target gene promoter region.

In our study, we first identified chr16:19884686-19885185 as the accessible chromatin region of the GPRC5B promoter through ATAC-seq profiles. Thereafter, we discovered that the mRNA expression of GATA4 was significantly associated with chr16:19884686-19885185. For confirmation, we acquired the ChIP-seq profiles of the GATA4 protein in colon cancer cells. The open chromatin region (chr16:19884686-19885185, highlighted in blue) was found to overlap with the GATA4 binding regions to a great extent. We also found that, with the upregulation and downregulation of GATA4 expression, GPRC5B would be regulated accordingly. Collectively, through this novel pipeline, we identified GATA4 as the direct upstream TF of GPRC5B.

Several inevitable limitations need to be addressed. First, our pipeline required paired ATAC-seq and RNA-seq profiles of the same samples, the amount of which was relatively small in public databases. However, despite the limited data sources, we acquired 41 eligible paired profiles to prove the effectiveness of our pipeline. Second, although we have identified GPRC5B as a key molecule in COAD prognosis through bioinformatics, we have not put forward any proof *in vivo*. Future studies should experimentally verify our findings.

Despite the limitations mentioned above, our study was the first to infer that GPRC5B, correlated with tumor-infiltrating macrophages, might be a key molecule affecting COAD prognosis. The interaction between GPRC5B and tumor-infiltrating macrophages could be a potential target for clinical therapy. We also proposed a novel pipeline, identifying GATA4 as a direct upstream TF of GPRC5B. This pipeline was based on the integration of multi-omics data, which was easy to apply and could be used to achieve a more convincing conclusion.

## Conclusions

GPRC5B, correlated with tumor-infiltrating macrophages, is a potential key molecule affecting COAD prognosis. Further, with our novel pipeline, TF GATA4 was identified as a direct upstream of GPRC5B. This study proposed a novel pipeline for TF exploration and provided a theoretical basis for COAD therapy.

## Data Availability Statement

Publicly available datasets were analyzed in this study. This data can be found here: https://portal.gdc.cancer.gov/, and https://gdc.cancer.gov/about-data/publications/ATACseq-AWG.

## Ethics Statement

The studies involving human participants were reviewed and approved by Tongji Hospital, Shanghai, China (reference number 2018-LCYJ-005). The patients/participants provided their written informed consent to participate in this study.

## Author Contributions

Conception/design: CH, ZN, BG, BD, and QH. Collection and/or assembly of data: CH, NZ, HX, ZN, XL, BL, BG, BD, and QH. Data analysis and interpretation: CH, HX, NZ, NW, ZN, BL, BD, and QH. Manuscript writing: CH, NW, ZC, and QH. All authors read and approved the final manuscript.

## Funding

This study was funded by Shanghai Science and Technology Innovation Action Plan (Grant No. 19441905700) and the Clinical Research and Cultivation Project of Shanghai Tongji Hospital (Grant No. ITJ (ZD) 1802, ITJ (ZD) 1804).

## Conflict of Interest

The authors declare that the research was conducted in the absence of any commercial or financial relationships that could be construed as a potential conflict of interest.

## Publisher’s Note

All claims expressed in this article are solely those of the authors and do not necessarily represent those of their affiliated organizations, or those of the publisher, the editors and the reviewers. Any product that may be evaluated in this article, or claim that may be made by its manufacturer, is not guaranteed or endorsed by the publisher.

## Abbreviations

TF, Transcription factor; qPCR, Quantitative polymerase chain reaction; COAD, Colon adenocarcinoma; GPCR, G protein-coupled receptor; ATAC-seq, Assay for transposase-accessible chromatin using sequencing; ChIP-seq, Chromatin immunoprecipitation sequencing; FPKM, Fragments per kilobase per million mapped reads; ssGSEA, Single Sample Gene Set Enrichment Analysis; TSS, Transcription start site; GEO, Gene Expression Omnibus; TCGA, The Cancer Genome Atlas; TPM, Transcripts per million.
