# Supplementary material for: Multi-Omics Analysis for Transcriptional Regulation of Immune-Related Targets Using Epigenetic Data: A New Research Direction
Source: Front Immunol. 2022 Jan 3;12:741634. doi: 10.3389/fimmu.2021.741634 (PMC8761734; doi:10.3389/fimmu.2021.741634)
Supplement: Supplementary file 1 [file Table_1.docx]

**Table S1** Baseline information of 426 patients diagnosed with colon adenocarcinoma.

| Variables | Total Patients (N = 426) |
| --- | --- |
| Age, years |  |
| Mean ± SD | 66.51 ± 12.76 |
| Median (Range) | 68 (31 - 90) |
| **Gender** |  |
| Female | 198 (46.48%) |
| Male | 228 (53.52%) |
| **Stage** |  |
| Stage I | 72 (16.90%) |
| Stage IA | 1 (0.23%) |
| Stage II | 28 (6.57%) |
| Stage IIA | 127 (29.82%) |
| Stage IIB | 9 (2.11%) |
| Stage IIC  Stage III | 1 (0.23%)  17(3.99%) |
| Stage IIIA | 6 (1.41%) |
| Stage IIIB | 57 (13.38%) |
| Stage IIIC | 39 (9.16%) |
| Stage IV  Stage IVA  Stage IVB | 41 (9.62%)  15 (3.53%)  2(0.47%) |
| unknow | 11 (2.58%) |
| **T** |  |
| T1 | 10 (2.35%) |
| T2 | 74(17.37%) |
| T3 | 291 (68.31%) |
| T4 | 24 (5.63%) |
| T4a | 18 (4.23%) |
| T4b | 8 (1.88%) |
| Tis | 1 (0.23%) |
| **N** |  |
| N0 | 253 (59.40%) |
| N1 | 69 (16.20%) |
| N1a  N1b  N1c  N2 | 15(3.52%)  13 (3.05%)  2 (0.47%)  53 (12.44%) |
| N2a  N2b | 8 (1.87%)  13 (3.05%) |
|  |  |
| **M** |  |
| M0 | 317 (74.41%) |
| M1 | 46 (10.80%) |
| M1a  M1b  MX  unknow | 9 (2.11%)  3(0.71%)  44(10.33%)  7(1.64%) |

**Abbreviations:** SD, Standard deviation.
